# Genome-Wide Association and Selective Sweep Analyses Reveal Candidate Genes Associated with Shoot Height in Maize Across Breeding Eras

**DOI:** 10.3390/genes17050570

**Published:** 2026-05-18

**Authors:** Junyue Wang, Baijuan Du

**Affiliations:** College of Agronomy, Shandong Agricultural University, Tai’an 271001, China; wangjunyue1995@163.com

**Keywords:** maize, seedling shoot height, genome-wide association study, selective sweep, breeding era

## Abstract

**Background:** Maize shoot height is an important component of early vigor and plant architecture; however, its genetic basis during seedling development and its relationship with modern breeding remain insufficiently understood. This study aimed to investigate the genetic architecture of maize seedling shoot height across different breeding eras. **Methods:** Shoot height at 21 days after sowing was evaluated in 363 maize inbred lines representing three breeding eras in China. Genome-wide association analysis was performed to identify loci and candidate genes associated with shoot height variation, and selective sweep analysis was used to detect breeding-era differentiated genomic regions. **Results:** Modern breeding lines from the 2000–2010s exhibited significantly greater shoot height than lines from earlier breeding periods. Pearson’s correlation analysis revealed that 3-week shoot height showed highly significant positive correlations with plant height and ear height. Selective sweep analysis identified multiple differentiated genomic regions harboring previously reported height- and architecture-related genes, including *ZmBR2*, *ZmLIL1*, *ZmNA1*, *ZmTE1*, *ZmSPL12*, *ZmBV1*, *ZmDIL1*, *ZmKN1* and *ZmACS7*. The GWAS identified 43 SNPs exceeding the GEC-derived suggestive threshold for shoot height, with the strongest and most continuous association signal located on chromosome 8. GWAS, together with LD analysis, haplotype analysis, and expression profiling, prioritized *ZmGDCL* (Zm00001d009163) as a promising candidate gene because of its strong association signal, local linkage disequilibrium support, broad expression profile, and significant haplotype effect on shoot height. **Conclusions:** Our results indicate that maize breeding has reshaped the genetic architecture of seedling shoot growth. *ZmGDCL* represents a promising candidate gene for future functional studies, while breeding-era differentiated regions provide useful genomic context for understanding maize architecture improvement.

## 1. Introduction

Maize (*Zea mays* L.) is one of the most important cereal crops worldwide and serves as a major source of food, feed, and industrial raw materials [1,2]. Sustained improvement of maize productivity remains essential for global food security under the combined pressures of population growth, climate change, and limited arable land [1]. In modern maize breeding, optimization of plant architecture is a major strategy for improving yield potential, and plant height is one of the core agronomic traits shaping canopy structure, light interception, lodging resistance, and planting density [3,4].

Plant height is a complex quantitative trait determined by coordinated developmental processes, including node formation, internode elongation, cell division, and cell expansion. Genetic studies have shown that maize height is highly heritable yet strongly polygenic, reflecting the combined contribution of many loci with small to moderate effects [5]. Numerous genes involved in hormone biosynthesis and signaling, meristem activity, and stem elongation have been reported to affect maize plant height and architecture, including *BR2*, *NA1*, *LIL1*, *TE1* and *ACS7* [6,7,8,9,10]. Recent genome-wide association studies and high-throughput phenotyping analyses have further revealed the complex genetic architecture underlying maize plant height and architectural traits, highlighting the contribution of multiple loci and developmental-stage-dependent regulatory networks to plant growth and breeding improvement [11,12,13,14]. Despite these advances, the genetic architecture of height-related traits remains incompletely understood, particularly in the context of long-term breeding selection and developmental-stage-specific variation.

Compared with adult plant height, shoot height at the seedling stage has received less attention, although early growth is agronomically important for seedling vigor, stand establishment, and adaptation to adverse environments. The seedling stage is a critical transition period during which maize shifts from dependence on seed reserves to active autotrophic growth. Rapid and uniform early shoot growth contributes to canopy establishment, efficient light interception, and subsequent biomass accumulation. Early vigor has also been widely recognized as an important determinant of stand establishment, growth uniformity, and productivity under suboptimal environments [15]. Because seedling shoot height reflects early vegetative growth potential and developmental performance, dissecting its genetic basis may help identify loci that contribute both to vigorous establishment and to subsequent architectural performance. Importantly, early shoot growth may also be closely linked to later plant architectural traits. In maize, plant height and ear height are major components of plant architecture that influence lodging resistance, canopy structure, and yield performance. Therefore, we hypothesized that natural genetic variation controlling seedling shoot height is associated with later plant architectural traits and that some of these loci may have been shaped by modern maize breeding. If shoot height at the seedling stage is significantly associated with later developmental traits, it may serve as an informative early indicator of subsequent plant growth and agronomic performance. Thus, dissecting the genetic basis of seedling shoot height is important not only for understanding early vegetative development but also for identifying loci potentially relevant to maize architecture improvement and breeding selection.

The genetic basis of maize height has been explored using both linkage mapping and genome-wide association studies. Earlier studies identified quantitative trait loci associated with plant and ear height, whereas later GWAS analyses further revealed numerous loci distributed across the maize genome and highlighted the polygenic nature of plant height regulation [5,16,17]. However, many associated loci remain insufficiently characterized, and most previous work has focused on adult plant height rather than seedling shoot height.

In parallel with association mapping, selection-scan approaches provide a powerful framework for identifying loci shaped by crop improvement. Previous genomic studies demonstrated that modern maize breeding has introduced substantial genome-wide changes and left abundant footprints of artificial selection in elite germplasm [4,18]. In parallel, selective sweep analysis can provide complementary evidence for identifying genomic regions shaped by breeding-associated differentiation.

In the present study, we investigated seedling shoot height in a maize association panel representing different breeding eras in China. We hypothesized that modern maize breeding has reshaped genetic variation associated with seedling shoot height and that combining association mapping with selective sweep analysis as complementary approaches could help identify candidate loci and breeding-associated genomic regions. Specifically, this study aimed to: (i) characterize variation in seedling shoot height across different maize breeding eras; (ii) identify genomic loci and candidate genes associated with seedling shoot height using GWAS; (iii) evaluate breeding-associated differentiated genomic regions using selective sweep analysis; and (iv) prioritize key GWAS candidate genes by integrating haplotype analysis, linkage disequilibrium information, and expression profiling.

## 2. Materials and Methods

### 2.1. Plant Materials and Phenotypic Evaluation

A maize association panel consisting of 363 inbred lines with broad genetic diversity was used in this study. According to the release year of each line and following the breeding-era framework reported by Ren et al. [19], the panel was divided into three breeding-era groups: CN1960&70s (China, 1960s–1970s), CN1980&90s (China, 1980s–1990s), and CN2000&10s (China, 2000s–2010s). The resequencing-based genotype resource used in this study was derived from the Zea variation dataset reported by Chen et al. [20]. This breeding-era classification was used for two complementary purposes. First, it allowed us to evaluate whether seedling shoot height changed across historical breeding periods, thereby assessing breeding-associated phenotypic changes over time. Second, it provided a population framework for selective sweep analysis, enabling us to identify genomic regions differentiated among breeding eras. These results were used as complementary evidence to interpret breeding-associated genomic changes related to maize shoot height and plant architecture. Seeds were germinated and grown in a greenhouse under controlled conditions. The daytime temperature was maintained at 25 ± 2 °C and the nighttime temperature at 20 ± 2 °C, with a relative humidity of 60–70% and a 14 h light/10 h dark photoperiod. Based on the recorded temperature and relative humidity, the daytime vapor pressure deficit (VPD) was estimated, rather than continuously monitored, to range from approximately 0.95 to 1.27 kPa. Plants were cultivated in plastic pots filled with a mixture of nutrient soil and perlite (3:1, *v*/*v*; Qingdao Keshang Biotechnology Co., Ltd., Qingdao, China). Soil water content was maintained at approximately 70% of field capacity throughout the experiment by regular watering. Shoot height was measured at 21 days after sowing. Six biological replicates were included for each line, and the mean value was used for subsequent analyses. The phenotyping experiment was conducted under a single greenhouse environment at one developmental stage. The phenotypic data are provided in Supplementary Table S1.

### 2.2. Selective Sweep Analysis

To investigate whether shoot height-related loci were subjected to breeding-associated differentiation across different historical periods, we performed selective sweep analysis based on population differentiation. The maize inbred lines were grouped according to breeding era, and pairwise comparisons were conducted among breeding-stage populations. Population differentiation was evaluated using the fixation index (Fst), which is widely used to assess genomic divergence between populations. Following the selective sweep strategy used by Ren et al. [19], a sliding-window approach was applied across the genome using a window size of 500 kb and a step size of 60 kb. Genomic regions with Fst values greater than or equal to 0.1 were retained as candidate differentiated regions. This threshold was selected because Fst values between 0.05 and 0.15 have been interpreted as indicating moderate genetic differentiation in maize population-genomic studies [21,22]. For example, Wegary et al. [21] used this classification in maize inbred lines and defined Fst values of 0.05–0.15 as moderate genetic differentiation, while Romay et al. [22] reported that an average Fst of 0.06 indicated moderate differentiation among major maize subpopulations. Therefore, Fst ≥ 0.1 was used as a preliminary cutoff to identify genomic regions showing appreciable allele-frequency differentiation among breeding-era groups. Previously reported shoot height- or plant architecture-related genes located within these candidate differentiated regions were further examined to evaluate their potential contribution to breeding-associated variation in shoot height.

### 2.3. Genome-Wide Association Study and Candidate Gene Identification

SNP markers were subjected to quality control using PLINK v2.0 [23]. Markers with a missing rate >= 20% or a minor allele frequency (MAF) <= 0.05 were excluded from further analysis. Genome-wide association analysis for shoot height was performed using EMMAX beta version 07Mar2010 with a mixed linear model, incorporating both population structure and relative kinship to control for confounding effects [24]. A total of 10,770,184 SNPs were used for the association analysis. The significance threshold was determined using the Genetic Type I Error Calculator (GEC v0.2) following Li et al. [25]. The GEC analysis estimated 2,413,339.57 effective independent markers from 10,770,184 observed SNPs (effective ratio = 0.22), corresponding to suggestive, significant, and highly significant thresholds of 4.14 × 10^−7^, 2.07 × 10^−8^, and 4.14 × 10^−10^, respectively. In this study, SNPs exceeding the suggestive threshold were regarded as suggestive association signals and were used for preliminary downstream candidate-gene screening, whereas SNPs exceeding the genome-wide significant threshold were considered genome-wide significant associations. Candidate genes were identified according to the genomic positions of associated SNPs and published LD decay information for maize inbred lines. Chen et al. reported genome-wide LD decay across the genus Zea and showed that LD in a diverse panel of maize inbred lines decayed to r^2^ = 0.1 at approximately 200 kb [20]. Therefore, genes located within 200 kb upstream or downstream of each associated SNP were considered candidate genes, using the maize B73 RefGen_v4 genome as the reference [20]. Functional annotation of candidate genes was obtained from MaizeGDB. Linkage disequilibrium (LD) analysis for the target region was performed using LDBlockShow v1.41 [26].

### 2.4. Expression Analysis of Candidate Genes

Because the chromosome 8 region harbored the strongest and most continuous association signal in the Manhattan plot, we focused downstream candidate-gene prioritization on this interval. By contrast, the signals on chromosome 1 were discontinuous, and no annotated genes were retrieved within the predefined candidate intervals for the loci on chromosomes 7 and 9. Therefore, expression patterns of genes located around the chromosome 8 peak were examined using publicly available ZEAMAP transcriptome data [27]. The selected expression profiles included the B73 primary root at 5 days, mature leaf, silk, and embryo at 38 days after pollination. Because these tissues do not fully match the seedling shoot stage used for shoot-height phenotyping, this expression analysis was used only to evaluate whether candidate genes were transcriptionally active in available maize tissues, particularly vegetative tissues, rather than as direct evidence for seedling shoot-height regulation.

### 2.5. Haplotype Analysis

For the major candidate gene region, SNPs exceeding the GEC-derived suggestive threshold and located within the local LD-supported interval were used for haplotype analysis. Accessions were grouped according to the allelic combinations of the associated variants, and shoot height differences among haplotypes were compared to evaluate the phenotypic effect of natural variation in the target locus.

### 2.6. Statistical Analysis

Phenotypic differences in shoot height among maize lines from different breeding eras were analyzed using one-way analysis of variance (one-way ANOVA). Before ANOVA, the assumptions of normality and homogeneity of variance were evaluated using the Shapiro–Wilk test, Q–Q plots, and Levene’s test. Shoot height data among breeding-era groups showed an approximately normal distribution and satisfied the assumptions required for ANOVA. Therefore, one-way ANOVA was used to compare shoot height among breeding eras.

Differences in shoot height between the major *ZmGDCL* haplotypes were assessed using Student’s *t*-test. Before the *t*-test, homogeneity of variance between haplotype groups was examined and confirmed, supporting the use of Student’s *t*-test rather than Welch’s *t*-test. All statistical analyses were performed in R v4.5.3. Statistical significance was defined as indicated in the corresponding figures.

## 3. Results

### 3.1. Shoot Height Varied Among Maize Lines from Different Breeding Eras and Was Closely Associated with Later Architectural Traits

To evaluate variation in early shoot growth across breeding periods, we measured shoot height in 363 maize inbred lines grown under greenhouse conditions. The panel was divided into three groups according to breeding era: CN1960&70s, CN1980&90s, and CN2000&10s. After 21 days of growth, substantial phenotypic variation in shoot height was observed across the association panel. One-way ANOVA revealed significant differences among breeding-era groups, with lines from the CN2000&10s group exhibiting significantly greater shoot height than those from the CN1960&70s and CN1980&90s groups.

**Figure 1:**
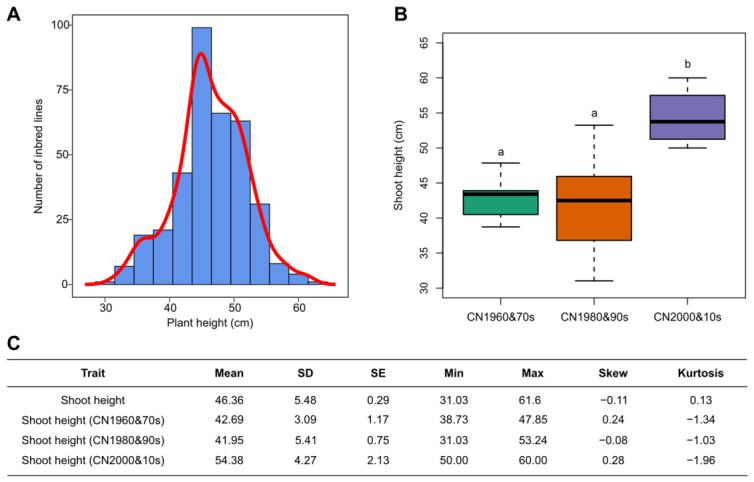
Phenotypic distribution and breeding-era comparison of 3-week shoot height in the maize association panel. (**A**) Frequency distribution of 3-week shoot height across 363 maize inbred lines, showing an approximately normal distribution. (**B**) Boxplot comparison of 3-week shoot height among breeding-era groups. Different letters indicate significant differences among groups. (**C**) Descriptive statistics of 3-week shoot height in the full panel and in each breeding-era group, including mean, standard deviation (SD), standard error (SE), minimum, maximum, skewness, and kurtosis.

Phenotypic distribution analysis further showed that 3-week shoot height ranged from 31.03 to 61.60 cm, with a mean value of 46.36 cm, a skewness of −0.11, and a kurtosis of 0.13, indicating an approximately normal distribution suitable for downstream association analysis (Figure 1A,C). Boxplot comparison among breeding-era groups showed that the CN2000&10s lines had significantly greater shoot height (54.38 cm) than the CN1960&70s (42.69 cm) and CN1980&90s (41.95 cm) groups, whereas no significant difference was detected between the two earlier groups (Figure 1B,C). These results indicate that modern breeding has been associated with a marked increase in seedling shoot height.

**Figure 2:**
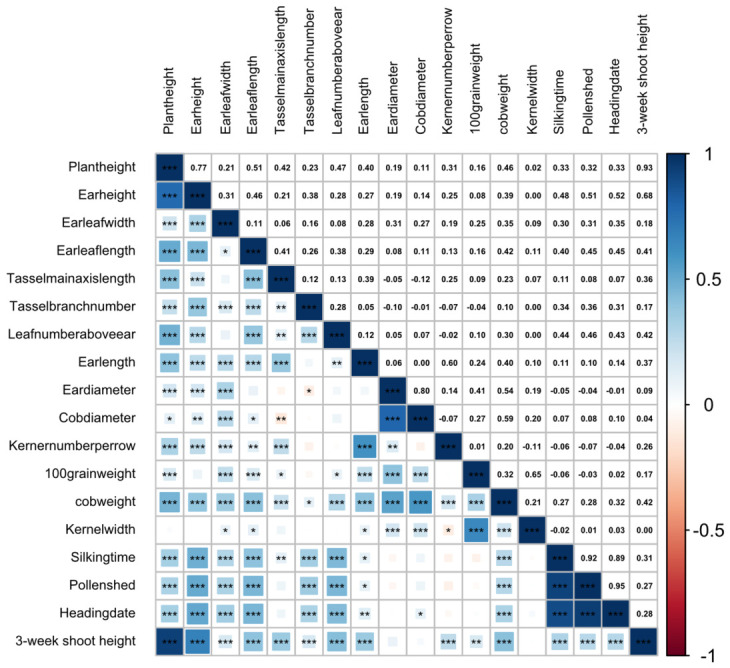
Pearson’s correlation analysis among 3-week shoot height and major agronomic traits in maize. The upper triangle shows Pearson’s correlation coefficients, and the lower triangle indicates correlation significance. Red and blue colors represent negative and positive correlations, respectively. Asterisks indicate significance levels (*, *p* ≤ 0.05; **, *p* ≤ 0.01; ***, *p* ≤ 0.001). Notably, 3-week shoot height showed a highly significant positive correlation with plant height (r = 0.93) and a highly significant positive correlation with ear height (r = 0.68), indicating that early shoot growth is closely associated with later plant architectural development.

To further assess the agronomic significance of seedling shoot height, we analyzed its correlations with other major traits. Pearson’s correlation analysis revealed that 3-week shoot height showed a highly significant positive correlation with plant height (r = 0.93, *p* ≤ 0.001) and a highly significant positive correlation with ear height (r = 0.68, *p* ≤ 0.001) (Figure 2). Because plant height and ear height are key components of maize architecture, these results indicate that early shoot growth is developmentally linked to later architectural traits. This finding further supports the biological importance of investigating shoot height at the seedling stage and suggests that seedling shoot height may serve as an informative early indicator of subsequent plant development.

### 3.2. Selective Sweep Analysis Identified Height-Related Genes Under Breeding Selection

To investigate whether shoot height-associated loci have been influenced by breeding selection, we performed selective sweep analysis across different breeding eras using population differentiation signals. This analysis identified multiple genomic regions showing evidence of selection, including several previously reported genes known to affect maize height or plant architecture. Among the genes detected in selected regions were *ZmBR2*, *ZmLIL1*, *ZmNA1*, *ZmTE1*, *ZmSPL12*, *ZmBV1*, *ZmDIL1*, *ZmKN1* and *ZmACS7*. These genes are functionally associated with hormone biosynthesis and signaling, meristem regulation, cell elongation, and internode development. In the comparison between CN2000&10s and CN1960&70s, several of these genes showed evidence of selection, including *ZmBR2*, *ZmLIL1*, *ZmSPL12*, *ZmBV1*, *ZmDIL1*, *ZmKN1* and *ZmACS7*. In the comparison between CN2000&10s and CN1980&90s, additional genes such as *ZmNA1* and *ZmTE1* were also detected. These findings suggest that modern breeding may have shaped multiple components of the genetic network underlying maize shoot height and plant architecture.

**Figure 3:**
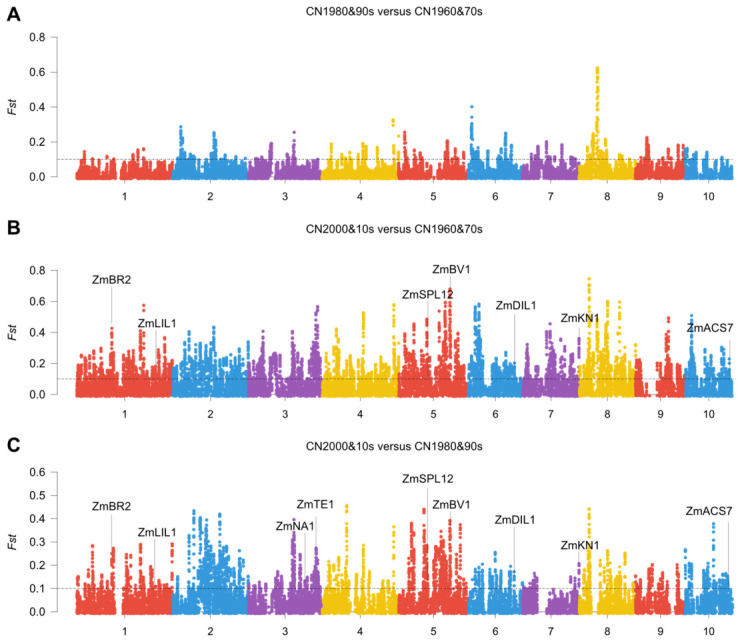
Genome-wide selective sweep analysis for shoot height-related loci across breeding eras. (**A**) Genome-wide Fst profile for CN1980&90s versus CN1960&70s. (**B**) Genome-wide Fst profile for CN2000&10s versus CN1960&70s. (**C**) Genome-wide Fst profile for CN2000&10s versus CN1980&90s. Different colors indicate different chromosomes or adjacent chromosomes in the genome-wide FST profiles. The dashed horizontal line indicates the threshold used to define selected regions (Fst = 0.1). Labeled peaks mark previously reported maize plant height- or architecture-related genes located within selected regions.

Genome-wide Fst scans showed comparatively weak differentiation between CN1980&90s and CN1960&70s, whereas both contrasts involving CN2000&10s displayed stronger and more frequent sweep signals across the genome (Figure 3A–C; Supplementary Table S2). In particular, the comparison between CN2000&10s and CN1960&70s highlighted pronounced peaks overlapping *ZmBR2*, *ZmLIL1*, *ZmSPL12*, *ZmBV1*, *ZmDIL1*, *ZmKN1* and *ZmACS7*, while the comparison between CN2000&10s and CN1980&90s additionally highlighted *ZmNA1* and *ZmTE1*. These patterns indicate that more recent breeding imposed stronger directional selection on genomic regions associated with maize plant architecture and shoot growth.

### 3.3. GWAS Identified Suggestive Association Signals for Shoot Height

**Figure 4:**
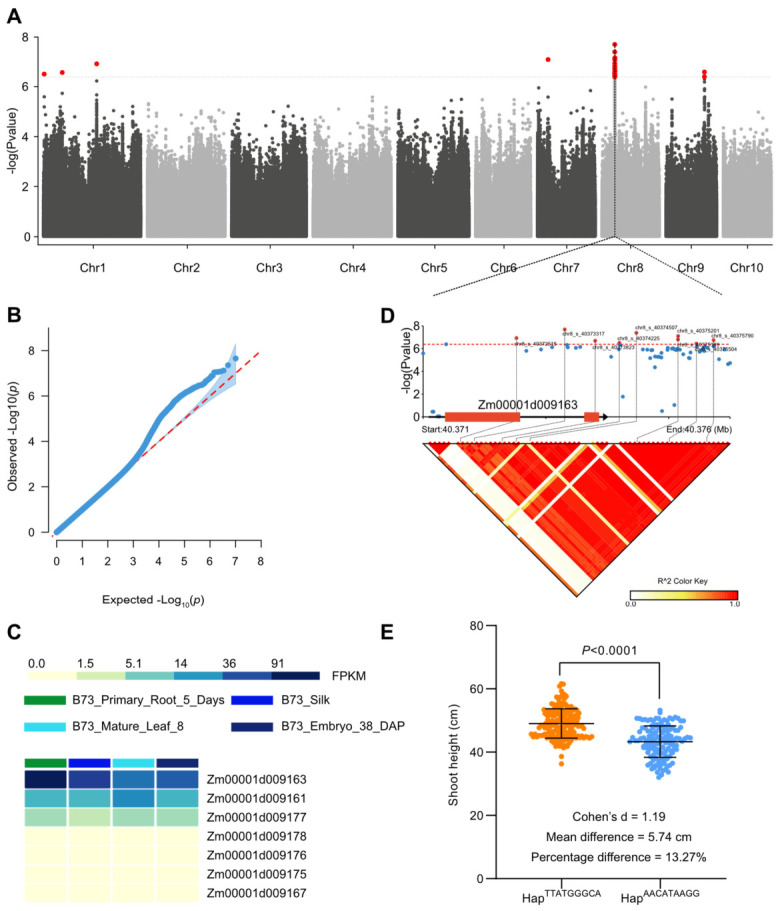
GWAS and candidate gene prioritization for 3-week shoot height in maize. (**A**) Manhattan plot of genome-wide association signals. The horizontal dashed line indicates the GEC-derived suggestive threshold used for association screening (−log10(*p*) = 6.38; *p* = 4.14 × 10^−7^), showing chromosome 8 as the major continuous association cluster together with additional discontinuous or sparse peaks on chromosomes 1, 7, and 9. (**B**) Quantile–quantile plot of observed versus expected −log10(*p*) values. The dotted diagonal line indicates the expected distribution under the null hypothesis. (**C**) Expression heatmap of genes located in the chromosome 8 candidate interval, based on publicly available ZEAMAP transcriptome data. The expression profiles were used to evaluate candidate-gene transcriptional activity across available maize tissues rather than as direct evidence of seedling shoot-height regulation. (**D**) Local association and linkage disequilibrium structure around the chromosome 8 peak containing *Zm00001d009163*. The upper panel shows local GWAS association signals, and the lower panel shows pairwise LD measured as r^2^. The dotted line in the upper panel indicates the GEC-derived suggestive significance threshold. The black triangular region indicates a strong local LD block containing the associated SNPs and overlapping *Zm00001d009163*, supporting chromosome 8 as a coherent candidate association region. (**E**) Haplotype comparison of 3-week shoot height between the two major *ZmGDCL* haplotypes. Boxplots show the distribution of shoot height for each haplotype, with individual maize lines shown as points. Lines carrying HapTTATGGGCA were significantly taller than those carrying HapAACATAAGG, with a mean difference of 5.74 cm, a 13.27% increase relative to HapAACATAAGG, and Cohen’s d = 1.19. Statistical significance was assessed using Student’s *t*-test.

To dissect the genetic architecture of shoot height, we performed a genome-wide association study using seedling-stage phenotypic data. As shown in the Manhattan plot, 43 SNPs exceeded the GEC-derived suggestive threshold of 4.14 × 10^−7^ (−log10(*p*) = 6.38) (Figure 4A). These signals were distributed across several chromosomes, but the chromosome 8 locus was clearly the strongest and most continuous association cluster. In contrast, the chromosome 1 signals were separated into discontinuous peaks, and the additional suggestive loci on chromosomes 7 and 9 were comparatively sparse. Using a predefined candidate-gene window of +/−200 kb around each associated SNP, annotated candidate genes were retrieved from the suggestive loci on chromosomes 1 and 8 (Table 1), indicating that shoot height is influenced by multiple loci but is dominated by a major associated region.

The quantile-quantile plot indicated that population structure and kinship were effectively controlled, with substantial deviation occurring mainly in the extreme tail of the distribution, consistent with the presence of true association signals rather than systematic inflation (Figure 4B). Because chromosome 8 represented the only major continuous peak and contained multiple annotated genes, we selected this region for expression profiling and fine-scale candidate prioritization. No annotated genes were identified within the predefined windows of the chromosome 7 and chromosome 9 signals, whereas the chromosome 1 associations remained discontinuous and therefore were not prioritized for downstream expression analysis. Local association and linkage disequilibrium analysis further delimited a strong candidate interval surrounding *ZmGDCL (Zm00001d009163)* within the chromosome 8 region (Figure 4D). Within this interval, *ZmGDCL (Zm00001d009163)* showed relatively high expression in representative maize tissues, including root, mature leaf, silk, and embryo-related samples, compared with neighboring genes in the same interval, further supporting its candidacy (Figure 4C). Consistent with this, haplotype comparison showed that HapTTATGGGCA was associated with significantly greater shoot height than HapAACATAAGG (*p* < 0.0001), supporting the contribution of natural variation at this locus to phenotypic differences in seedling shoot height (Figure 4E; Supplementary Table S3).

### 3.4. Integrative Analyses and Haplotype Comparison Prioritized ZmGDCL as a Candidate Gene

To further evaluate candidate genes in the major associated region highlighted by the GWAS, we examined expression patterns of genes in the chromosome 8 interval, because this region showed the strongest and most continuous association signal. The chromosome 1 signals were discontinuous, and no annotated genes were retrieved for the loci on chromosomes 7 and 9 within the predefined candidate windows.

To further assess the phenotypic relevance of sequence variation in the *ZmGDCL* region, we carried out haplotype analysis and identified two major haplotypes. Student’s *t*-test showed that HapTTATGGGCA was associated with significantly greater 3-week shoot height than HapAACATAAGG. Lines carrying HapTTATGGGCA showed a 5.74 cm higher mean shoot height than those carrying HapAACATAAGG, corresponding to a 13.27% increase. The effect size was large, as indicated by Cohen’s d = 1.19, suggesting a pronounced haplotype-associated effect on shoot height.

These results indicate that natural variation in the *ZmGDCL* locus is associated with measurable differences in maize seedling shoot height. Taken together, the strong GWAS signal, local LD pattern, haplotype effect, and expression evidence from available maize tissues support *ZmGDCL* as a priority candidate gene for shoot-height variation. More broadly, GWAS and selective sweep analyses provided complementary evidence for understanding the genetic basis of maize shoot height variation and breeding-associated genomic differentiation.

## 4. Discussion

In this study, we combined GWAS, selective sweep analysis, haplotype analysis, and expression profiling to investigate the genetic basis of shoot height variation in maize across different breeding eras. Our results revealed clear phenotypic differences among breeding-era groups and showed that modern maize lines exhibited significantly greater shoot height than earlier lines. These observations suggest that maize breeding has altered the genetic architecture of early shoot growth and that some shoot height-associated loci may have been shaped by artificial selection during modern improvement.

The highly significant positive correlations of 3-week shoot height with plant height and ear height further emphasize the developmental and agronomic relevance of this early-stage trait. Because both plant height and ear height are key determinants of maize architecture, the close relationship observed here suggests that seedling shoot height captures important aspects of the developmental program underlying final plant stature. This also supports the use of seedling shoot height as a meaningful target trait for genetic dissection and breeding-oriented analysis.

Shoot height is an important component of maize architecture and is closely associated with seedling vigor, early establishment, canopy development, and subsequent agronomic performance. Therefore, identifying loci associated with seedling shoot height is important not only for understanding developmental regulation, but also for improving crop establishment and the optimization of plant architecture in breeding programs [4,15].

The approximately normal distribution of 3-week shoot height and the clear phenotypic divergence among breeding-era groups further support the suitability of this trait for population-level genetic dissection and suggest that breeding-era differences are consistent with an increase in seedling shoot height during modern maize improvement.

The selective sweep analysis further supports the contribution of breeding to shoot height variation. We detected multiple previously reported genes associated with plant height and architecture in regions showing breeding-associated differentiation, including *ZmBR2*, *ZmLIL1*, *ZmNA1*, *ZmTE1* and *ZmACS7*. These genes are known to participate in hormone-mediated growth regulation, meristem activity, and internode elongation, indicating that modern breeding has likely acted on several interconnected developmental pathways rather than on a single locus [6,7,8,9,10]. The detection of known height-related genes in selected regions also increases confidence in the biological relevance of our population-level analysis.

Our GWAS results identified 43 suggestive association signals for shoot height. Among them, the chromosome 8 locus stood out as the strongest and most continuous association signal, whereas the chromosome 1 associations were discontinuous and the signals on chromosomes 7 and 9 were comparatively sparse. This pattern, together with the absence of annotated genes in the predefined windows on chromosomes 7 and 9, supported the prioritization of the chromosome 8 region for downstream candidate-gene analysis. Among the candidate genes in this region, *ZmGDCL* was prioritized because it showed the strongest statistical association, relatively high expression across multiple tissues, and significant haplotype variation associated with shoot height. These lines of evidence support *ZmGDCL* as a promising candidate gene associated with natural variation in maize seedling shoot growth.

*ZmGDCL* (*Zm00001d009163*) is annotated as a dihydrolipoyl dehydrogenase or glycine decarboxylase (GDC) L-protein-related gene. Based on published comparisons of plant GDC gene families, its candidate homologs include the Arabidopsis mitochondrial lipoamide dehydrogenase genes *LPD1* (*AT1G48030*) and *LPD2* (*AT3G17240*), as well as the rice GDC L-protein-related genes *LOC_Os01g22520* and *LOC_Os05g06750* [28,29]. Previous studies have shown that overexpression of the GDC H-protein enhances photosynthesis and growth in Arabidopsis, whereas knockdown of rice GDCH causes severe photorespiratory defects, growth inhibition, and senescence-associated phenotypes [30,31,32]. These reports refer to the H-subunit rather than the L-protein-related gene represented by *ZmGDCL*; nevertheless, they indicate that proper GDC complex function is essential for normal plant growth. In addition, reduced mitochondrial dihydrolipoyl dehydrogenase activity in Arabidopsis alters photorespiration and photosynthetic performance, further supporting the idea that *ZmGDCL* may influence shoot height through growth-related metabolic regulation [33]. Although these studies were not conducted on maize *ZmGDCL* itself and therefore cannot be taken as direct functional evidence, they support the biological plausibility that variation in a GDC L-protein-related gene may influence seedling growth through effects on mitochondrial metabolism, photorespiration, and carbon–nitrogen balance.

More importantly, our study highlights the value of using association mapping and breeding-selection scans as complementary approaches to investigate complex agronomic traits. Compared with GWAS alone, complementary selective sweep analysis can provide additional context for interpreting whether height- or architecture-related genomic regions may have been influenced by crop improvement history. Similar breeding-era genomic studies in maize have shown that historical sampling can effectively reveal selected loci and favorable alleles shaped during modern improvement [4,18].

Several limitations of the present study should also be acknowledged. First, the phenotyping experiment was conducted under a single controlled greenhouse environment and at one developmental stage, namely 21 days after sowing. For each inbred line, six individual plants were measured as biological replicates, and the mean value was used for downstream analyses; however, the experiment was not independently repeated across multiple environments or seasons. The greenhouse conditions were controlled, with a daytime temperature of 25 ± 2 °C, a nighttime temperature of 20 ± 2 °C, a relative humidity of 60–70%, a 14 h light/10 h dark photoperiod, and an estimated daytime vapor pressure deficit (VPD) of approximately 0.95–1.27 kPa. Soil water content was maintained at approximately 70% of field capacity. Nevertheless, this single-environment and single-time-point design limits our ability to evaluate genotype-by-environment interactions and developmental-stage-specific genetic effects. Shoot height and plant architecture are complex quantitative traits that may be influenced by temperature, light intensity, humidity, soil water availability, VPD, and other environmental factors. Therefore, some association signals or candidate loci detected in this study may be specific to the tested greenhouse conditions or seedling stage. Future multi-environment trials, repeated-season experiments, and phenotyping across additional developmental stages will be necessary to evaluate the stability of these loci and to validate the contribution of candidate genes such as *ZmGDCL* to maize shoot-height variation. Second, although multiple candidate genes were prioritized through the combined analyses, their causal variants and molecular mechanisms remain unresolved. Fine-mapping and functional analyses will therefore be required to establish the biological roles of the prioritized genes.

Overall, our study reveals that shoot height variation in maize has been shaped by breeding-associated genetic changes and provides a set of promising candidate genes for future dissection of maize architecture. These findings expand our understanding of the genetic basis of seedling shoot height and provide useful resources for crop improvement.

## 5. Conclusions

In conclusion, this study integrated phenotypic evaluation, genome-wide association analysis, selective sweep analysis, haplotype analysis, and expression profiling to dissect the genetic basis of maize seedling shoot height across breeding eras. By classifying maize inbred lines according to their release years, we showed that seedling shoot height differed significantly among historical breeding periods, with modern lines from the 2000–2010s exhibiting greater shoot height than earlier lines. This result suggests that early shoot growth has changed during modern maize improvement and may reflect breeding-associated shifts in plant developmental and architectural traits.

The highly significant positive correlations between seedling shoot height and later plant architectural traits, particularly plant height and ear height, further highlight the biological and agronomic relevance of this early-stage trait. These findings indicate that seedling shoot height is not only a measure of early vigor but may also serve as an informative indicator of subsequent plant development. Therefore, identifying loci associated with seedling shoot height can contribute to a better understanding of maize growth regulation and may provide useful targets for breeding programs aimed at improving early establishment and plant architecture.

Through GWAS, we identified multiple loci associated with shoot height, with the strongest and most continuous association signal located on chromosome 8. By integrating association signals with LD structure, haplotype effects, and expression profiles, we prioritized *ZmGDCL* as a promising candidate gene underlying natural variation in maize seedling shoot height. In addition, selective sweep analysis identified several known height- and architecture-related genes in differentiated genomic regions, supporting the idea that modern breeding has shaped multiple components of the genetic network controlling maize growth. Overall, this study provides new insight into the genetic basis of seedling shoot height and its relationship with maize breeding history. The candidate loci identified here, especially *ZmGDCL*, represent valuable targets for future fine-mapping, functional validation, and molecular breeding.

## Figures and Tables

**Table 1:** Candidate genes identified around SNPs exceeding the GEC-derived suggestive threshold for shoot height.

SNP Marker	Chr	Position	*p*-Value	−log10(*p*)	PVE	Candidate Interval	Candidate Genes	Gene Positions	Distance from SNP to Gene	Description
chr1.s_2157220	Chr1	2,157,220	3.165 × 10^−7^	6.50	0.07296	Chr1: 1,957,220–2,357,220	Zm00001d027298 Zm00001d027299	Zm00001d027298: Chr1: 2,154,232–2,156,387 Zm00001d027299: Chr1: 2,157,230–2,170,332	833 bp 10 bp	Bestrophin polymerase II transcription-mediator1
chr1.s_59030407	Chr1	59,030,407	2.741 × 10^−7^	6.56	0.06508	Chr1: 58,830,407–59,230,407	Zm00001d029122 Zm00001d029123 Zm00001d029124	Zm00001d029122: Chr1: 59,021,967–59,022,404 Zm00001d029123: Chr1: 59,023,014–59,027,798 Zm00001d029124: Chr1: 59,028,004–59,030,626	8003 bp 2609 bp within gene	Rapid alkalinization factor15 Repair protein Rad1/Rec1/Rad17 40S ribosomal protein S21b
chr8.s_40272513	Chr8	40,272,513	2.682 × 10^−7^	6.57	0.08195	Chr8: 40,072,513–40,472,513	Zm00001d009161	Zm00001d009161: Chr8: 40,267,885–40,272,449	64 bp	Bucentaur or craniofacial development
chr8.s_40373317	Chr8	40,373,317	2.060 × 10^−8^	7.69	0.08910	Chr8: 40,173,317–40,573,317	Zm00001d009163	Zm00001d009163: Chr8: 40,371,057–40,374,042	within gene	Gly decarboxylase L-protein
chr8.s_40618071	Chr8	40,618,071	8.199 × 10^−8^	7.09	0.08220	Chr8: 40,418,071–40,818,071	Zm00001d009167	Zm00001d009167: Chr8: 40,630,094–40,633,047	12,023 bp	polygalacturonase51
chr8.s_41013533	Chr8	41,013,533	4.000 × 10^−7^	6.40	0.07240	Chr8: 40,813,533–41,213,533	Zm00001d009175 Zm00001d009176	Zm00001d009175: Chr8: 41,010,962–41,012,858 Zm00001d009176: Chr8: 41,015,133–41,016,293	675 bp 1600 bp	Uncharacterized protein Leucine Rich Repeat
chr8.s_41081959	Chr8	41,081,959	2.370 × 10^−7^	6.63	0.07505	Chr8: 40,881,959–41,281,959	Zm00001d009177 Zm00001d009178	Zm00001d009177: Chr8: 41,086,584–41,090,905 Zm00001d009178: Chr8: 41,092,695–41,101,111	4625 bp 10,736 bp	invertase alkaline neutral9 Serine carboxypeptidase-like 33

## Data Availability

The data supporting the findings of this study are available within the article and its Supplementary Materials. The resequencing-derived genotype dataset analyzed in this study was obtained from the published Zea variation resource cited in the manuscript.

## Supplementary Materials

The following supporting information can be downloaded at: https://www.mdpi.com/article/10.3390/genes17050570/s1. Table S1: phenotypic data for the maize association panel; Table S2: selective sweep candidate regions and height-related genes across breeding-era comparisons; Table S3: haplotype information of *ZmGDCL*.
